# Effectiveness of stress management training on the psychological well-being of the nurses

**Published:** 2015

**Authors:** M Pahlevani, M Ebrahimi, S Radmehr, F Amini, M Bahraminasab, M Yazdani

**Affiliations:** * Master of Clinical Psychology, Islamic Azad University of Ayatollah Amoli Branch, Amol, Iran; ** Master of Clinical Psychology, Islamic Azad University, Science and Research Branch, Tehran, Iran; *** Master of Clinical Psychology, Islamic Azad University Chaloos Branch, Iran; **** Master of Educational Psychology, Islamic Azad University Central Tehran Branch, Iran; ***** Master of Educational Management, Islamic Azad University Garmsar Branch, Iran; ****** Master of General Psychology, Payame Noor University, South Tehran Branch, Iran

**Keywords:** stress management, psychological well-being, nurses

## Abstract

**Objective:** an appropriate psychological intervention to promote the level of the public health and mental well-being of nurses has a great importance. This investigation was aimed to study the effectiveness of stress management training on the psychological welfare of nurses in Imam Khomeini Hospital.

**Methodology:** this study was quasi-experimental with pretest-posttest that used a control group. Hence, 40 of the nurses in Imam Khomeini Hospital were selected by using a convenience sampling method and placed in the experimental group and the control group. Both groups were pretested by using psychological well-being 84-question scale. Afterwards, the experimental group was trained for ten sessions under stress management skill exercise, and the check group got no intervention. Next, both societies were post-tested, and the acquired data were analyzed by using inferential and descriptive statistical methods accompanied by SPSS 21 software.

**Findings:** the results indicated that stress management training significantly led to the promotion of psychological well-being in nurses (p < 0.001).

**Conclusion:** it was found from the research that due to the high level of effectiveness of stress management training, its low cost, and its high acceptability by the patients, especially when it was performed in a group, had a significant positive impact on the promotion of psychological well-being in nurses.

## Introduction

The nursing service is at the heart of the health care systems, so, nurses have the most quantity of individuals working in the health sectors. Moreover, due to the direct relationship of nurses with the patients, this profession has a significant level of importance [**1**]. A nurse is a person who is responsible for the twenty-four-hour monitoring of patients, thereby he/ she is continually exposed to stressful factors [**2**]. There have been many types of research indicating that nursing is among the professions that are influenced by the negative impacts of physical and psychological stresses caused by the occupational activities [**1**]. Therefore, it is important to conduct a study to improve their conditions.

Psychological well-being is a vital and influencing psychological factor in the improvement of working conditions and personal life of nurses [**3**]. Well-being is divided into two types, psychological well-being, and subjective well-being. Mental well-being is defined as the growing of real talents of each and consists of six components of goal in life, positive relationships with others, personal growth, self-acceptance, autonomy and environmental mastery [**4**]. The purpose in life means having a purpose and direction in life and following those objectives [**5**]. Having positive relations with others means having warm, satisfactory relationships together with insurance and empathy [**6**]. Personal growth refers to having a continuous sense of growth and sense of efficacy and increasing knowledge [4]. Environmental mastery means the feeling of competence and ability to manage complicated environments [**5**]. Moreover, a subjective well-being included the way one’s evaluation of his/ her life and consisted of two dimensions, cognitive and emotional. Cognitive components include life satisfaction and field satisfaction and emotional components include positive and negative emotions [**7**].

Many types of research indicated that life occurrences and events could influence psychological well-being. In other words, the long-term facing with unpleasant incidents and situations of life could affect the mental well-being and could impair it, thereby several psychological problems emerging [**8**]. The working condition is one of the most important events that could affect several life aspects and psychological well-being. In addition, nurses face a broad spectrum of stressful factors such as confronting with severe disease, the death of patients, enormous amounts of work and role ambiguity. It could have a vital role in their work [**9**], and change to a source of stress and endanger their physical and psychological health. It is while nurses are regularly concerned and interested in people when they start this career. However, most of them feel exhausted after a period of working and the exposure to a host of problems and job stress. They even prefer to quit their job [**2**]. According to the fact that psychological well-being plays a major role in the individuals’ work life and personal life, conducting such a study seems to be significant.

In the meantime, life skills training play a major role in one’s psychological well-being and health [**10**]. According to UNICEF (2011) [**11**], life skills refer to an approach based on a change the in behavior or forming behavior which is considered balancing between the three areas of knowledge, attitude, and skills. WHO [**12**] defines life skill as an ability that leads to the promotion of the individuals’ mental health in a community, enrichment of human relationships, enhancement of health and healthy behaviors in the society. Moreover, this organization introduced ten skills of decision-making, problem-solving, creative thinking, critical thinking, effective communication, interpersonal connection abilities, and self-awareness, empathy, coping with emotions and dealing with stress as primary life skills.

Work stress is the major problem of nurses, which has a high impact on their professional performance and personal life. It sometimes leads to a severe conflict between labor and life; it seems that stress management skills training results in the promotion of the psychological well-being. During stress management skills training, the person is taught to program for their life affairs and studying, setting a reasonable schedule and preparing himself for unpredictable issues [**13**]. Therefore, paying a particular attention to such training in nursing is important due to stressful conditions in this career. While studying the literature review in Iran it was indicated that, there had been no research on the effectiveness of stress management skills training on the psychological well-being of nurses. Hence, conducting a precise study on this subject seems to be necessary.

Therefore, resolving this research gap in Iran and the necessity of promoting the psychological well-being of nurses by conducting such a study was deemed necessary. According to what has been said, the present research aimed to investigate the effectiveness of stress management training on the psychological well-being of nurses in Imam Khomeini Hospital.

## Methodology

The current study was quasi-experimental with a pretest-posttest that used a control group. The population consisted of all the nurses employed in Imam Khomeini Hospital in 2015, who were selected by using a convenience sampling method. Based on the fact that the least sample of people in the empirical investigations had to be of 15 individuals, a 15-individuals sample size was chosen for each of the groups [**14**]. Afterwards, to boost the statistical power and to control the decreased number of participants, a 20-individuals sample size (n = 20) was chosen for each of the groups. Sampling was carried out among all the nurses employed in Imam Khomeini Hospital based on a voluntary and non-random method. The formation index of this research was the informed comfort and the willingness to partake in the research, experience to take part in sessions and cooperation in doing assignments. Also, it included a desire to cooperate in completing the instruments, the age range between 20 and 45, having the minimum education level of diploma and having the physical and psychological stability to take part in the research. The exclusion criteria for the research were the lack of willingness to participate in the investigation and the loss of more than three sessions in the practiced manner. Also, it included the lack of experience to take part in the sessions and the lack of experience to cooperate in doing the assignments and receiving any kind of training and psychological treatment out of the schedule and program set in the research.

The implementation method meant that sampling was conducted of all the nurses employed in Imam Khomeini Hospital randomly and voluntarily. The selected sample size was then placed into two groups of experimental and control in case that they had the required inclusion criteria. Prior to the implementation of the research, in order to observe the ethical principles and to ensure the attendance to meetings, informed consents were obtained in addition to explaining about the investigation and its positive impacts, being assured that the received data would remain confidential. Then, the experimental group was trained for ten sessions under stress management skills training and the control group received no intervention. Finally, both groups were post-tested. The employed protocols for the behavioral-cognitive group therapy sessions are mentioned in **Table 1**. 

The instruments used in the present research consisted of a Psychological well-being scale and a Demographic Research Paper.

Demographic Research Paper: the demographic research paper included age, gender, education level, primary and marital status. This article was provided and evaluated by the related scholars and researchers.

The Scale of Psychological Well-Being (SPWB): the Scale of Psychological Well-Being of Sirigatti S, Penzo I, Giannetti E, Casale S, Stefanile C (2016) [**8**] consisted of 84 questions based on a 7-point Likert scale (from “entirely disagree” to “agree absolutely”). This scale was of self-report scale which evaluated six components of the psychological well-being including a goal in life, certain connections with others, personal extension, self-acceptance, autonomy and environmental mastery. The internal consistency coefficients for the mentioned components were from 0.83 to 0.91. According to Joshanlou and Mohammadpour (2014), the reliability of the scale was obtained as being equal to 0.81 for the scale of psychological well-being, by using Cronbach’s alpha method. This coefficient was equal to 0.60, 0.64, 0.54, 0.58, 0.65, and 0.61 for the subscales of autonomy, environmental mastery, personal growth, positive relations with others, purpose in life and self-acceptance. In a research conducted by Kafka and Kuzma [**15**], which investigated the validity of Ryff psychological well-being scale, findings indicated that there was an excellent correlation between the mentioned scale and the subjective psychological well-being (SWB) and the scale of life satisfaction (SWLS). Using Cronbach’s alpha method, the present research found the value of 0.81 for the scale of psychological well-being and the values of 0.60, 0.64, 0.54, 0.58, 0.65, and 0.61 for the subscales of autonomy, environmental mastery, personal growth, positive relations with others, purpose in life and self-acceptance.

Statistical Package for Social Sciences (SPSS-21) software was used to analyze the obtained data. To analyze the research data, based on descriptive statistics, indices of average, standard deviation, frequency, and frequency percentage were used. Moreover, based on inferential and statistics, one variable and multivariable Analysis of Covariance (ANCOVA) was used.

**Table 1:** Cognitive-behavioral group therapy training protocol

Session	Subject
First	Referral of group members, practicing to get acquainted, introducing stress, stressors and stress responds, and understanding of physical responses related to stressors
Second	Making them aware of the effects of stress and making them understand the importance of this awareness and increasing the awareness about physical responses related to stressors
Third	Explaining the relationship between thoughts, emotions and physical sensations and offering numerous examples in various situations
Fourth	Introducing and identifying common types of negative thoughts and cognitive distortions
Fifth	Providing challenge in the field of negative common thoughts and cognitive distortions and replacing rational thoughts with irrational thoughts
Sixth	Training, practicing and implementing effective coping strategies
Seventh	Continuing training, practicing and implementing effective coping strategies
Eighth	Training and discussing about anger management, assertiveness, time management, recording daily events
Ninth	Using the skills of problem solving regarding conflicts, discussing the skills to say no, delegation
Tenth	Training the importance and understanding the benefits of social protection and an overview of the program

**Research Findings**

The demographic characteristics of the present sample size are listed in **Table 2**.

**Table 2:** Demographic characteristics of the subjects

Variable	Group	Frequency	Frequency percentage	Average and standard deviation
Age	25-30	11	27.5	33.25 ± 5.39
	31-35	14	35	
	36-40	9	22.5	
	41-45	6	15	
Level of education	Bachelor degree	33	82.5	
	Master degree an above	7	17.5	
Marital status	Bachelor	37	92.5	
	Married	3	7.5	

As demonstrated in **Table 2**, the most frequent participants were dedicated to individuals between the age of 31 and 35 years old (14 persons or 35%) and the least frequent participants were assigned to people between the age of 41 and 45 years old (6 individuals or 15%). In addition, the average age of the participants was 33.25 and, the standard deviation was 5.39. Other demographic information was mentioned in **Table 2**.

**Table 3:** Descriptive statistics of scores of psychological well-being in the two groups according to the pretest and post test

Component	Index	Experiment		Control	
		Pretest	Posttest	Pretest	Posttest
Purpose in life	Average	47.80	57.65	49.35	50.05
	Standard deviation	6.46	7.89	5.17	4.97
Positive relations with others	Average	51.70	61.95	49.50	50.25
	Standard deviation	5.86	7.07	3.41	3.95
Personal growth	Average	51.60	59.95	51.40	50.65
	Standard deviation	4.17	4.51	2.78	2.30
Self-acceptance	Average	48.59	59.30	49.35	49.60
	Standard deviation	3.15	3.90	4.20	4.10
Autonomy	Average	46.80	56.70	48.50	47.70
	Standard deviation	3.03	4.07	3.56	3.49
Environmental mastery	Average	48.15	56.80	47.45	47.40
	Standard deviation	5.15	5.03	5.06	5.45
Psychological well-being	Average	295.01	325.35	295.55	295.65
	Standard deviation	13.68	15.46	11.65	12.09

According to **Table 3**, the average scores of the variables of purpose in life, positive relations with others, personal growth, self-acceptance, autonomy, environmental mastery and psychological well-being in the stage of posttest increased in the experimental group compared to the control group.

**Table 4:** Levene test results which investigate the default homogeneity of variances of physical, emotional and social dimensions in posttest

Variable	Stage	F	Df1	Df2	Sig. level
Purpose in life	Posttest	2.593	1	38	0.116
Positive relations with others	Posttest	1.652	1	38	0.206
Personal growth	Posttest	3.021	1	38	0.090
Self-acceptance	Posttest	0.434	1	38	0.514
Autonomy	Posttest	0.241	1	38	1.418
Environmental mastery	Posttest	0.015	1	38	0.903
Psychological well-being	Posttest	0.702	1	38	0.408

According to **Table 4**, the null hypothesis of equality of variances of the two groups in a variable of psychological well-being and its components were approved. In other words, the variances of the two groups were equal to each other for the variable of mental well-being and its components, and there was no significant difference. Therefore, according to the compliance with the Levine defaults, the results of the study research hypotheses were permissible.

**Table 5:** Results of multivariable ANACOVA on the scores of posttest with control of pretest in the variable of psychological well-being and its components

Test	Value	F	Df	Sig. level	Squared Eta	Power
Pylayy effect	0.894	46.287	6	0.001	0.894	0.95
Wilks Lambda	0.106	46.287	6	0.001	0.894	0.95
Hotelling effect	8.416	46.287	6	0.001	0.894	0.95
Ray’s largest root	8.416	46.287	6	0.001	0.894	0.95

As mentioned in **Table 5**, the significance level of all the tests (P < 0.001) revealed that there were variations between the two groups in at least one of the dependent variables (psychological well-being and its components). Based on the squared eta, 0.89 percent of the observed differences between individuals was related to the impact of the independent variable (i.e. intervention method). On the other hand, since the statistical power was equal to 0.95 (greater than 0.80), the sample size was admissible. The results related to the significant difference of each of the dependent variables were mentioned in the following table.

**Table 6:** Results of multivariable ANACOVA in order to investigate the effectiveness of stress management skills training on the psychological well-being and its components in the posttest

Index	Sum of squares	Df	Mean Square	F	Sig. level	Squared Eta
Purpose in life	577.600	1	577.600	13.258	0.001	0.259
Positive relations with others	1368.900	1	1368.900	42.267	0.001	0.527
Personal growth	864.901	1	864.901	67.418	0.001	0.640
Self-acceptance	940.900	1	940.900	58.518	0.001	0.606
Autonomy	810.000	1	810.000	56.127	0.001	0.596
Environmental mastery	883.600	1	883.600	32.100	0.001	0.458
Psychological well-being	32148.900	1	32148.900	166.823	0.001	0.814

Based on **Table 6**, since p < 0.001, the hypothesis related to the differences between psychological well-being and its components between the two groups is approved. Moreover, it can be expressed that 0.25, 0.52, 0.64, 0.60, 0.59, 0.45 and 0.81 percent of the change in the score of the purpose of life, a positive relationship with others, personal growth, self-acceptance, autonomy, environmental mastery and psychological well-being were due to the independent variable. Therefore, it could be expressed that stress management skills training led to an increase of mental well-being and its components in nurses.

## Conclusion

Since the present research was conducted to study the effectiveness of stress management skills training on the psychological welfare of nurses in Imam Khomeini Hospital, the findings obtained from single variable and multivariable ANACOVA indicated that stress management skills training had a significant impact on the psychological welfare of nurses. The findings were in line with the previously conducted studies such as the ones of Alavi Arjomand N, Kashani Nia Z, Hosseini MA, Reza Soltani P (2012), Ghadiri Bahram Abadi F, Mikaili F (2015), Ghanbari N, Habibi M, Shams Al-Dini S (2013), Chubforoushzadeh A, Kalantari M, Molavi H (2009) [**13**,**16**-**18**]. 

According to Ghadiri Bahram Abadi F, Mikaili F (2015) [**16**], a continuous exposure to multiple stress demanded training and learning appropriate skills and techniques to cope with stress. In other words, facing with stress, individuals should have the necessary and required coping skills to reduce or eliminate it. The person can deal with life issues and challenges better when he/ she can cope with stress and manage it. Therefore, stress management intervention leads to the formation of healthy and desirable feelings in one’s life and thereby his/ her self-confidence increases. As a result, there would be an increase in the level of psychological well-being.

Ghanbari N, Habibi M, Shams Al-Dini S (2013) [**17**] found in their research that tension and anxiety were reduced by using a variety of coping with stress strategies such as relaxation. Moreover, individuals identified physical symptoms and reduced undesirable feelings and anxiety by mastering in relaxation, which was inconsistent with tension. As a result, their psychological well-being increased. Chubforoushzadeh A, Kalantari M, Molavi H (2009) [**18**] also deduced that stress management treatments make a variety of changes in one’s beliefs, feelings, and behaviors. Therefore, the promotion of the person’s evaluation and the improvement of his/ her coping skills and presented practices to integrate learned separations with real life situations led to a reduction of the perceived stress and increased psychological well-being.

**Acknowledgement**

The authors would like to thank the respected authorities of Imam Khomeini Hospital for their assistance. Moreover, the authors would also like to thank all the nurses for their cooperation in the present research.
